# The role of sex, gender, and SSDH in resilience research

**DOI:** 10.1002/alz70860_099970

**Published:** 2025-12-23

**Authors:** Gillian Einstein

**Affiliations:** ^1^ University of Toronto, Toronto, ON, Canada

## Abstract

**Background:**

When approaching a new research area such as risk and resilience in diverse underrepresented communities, it can be enlightening to turn to an area of diversity in which much research has already been conducted: sex differences, gender, and women's health. Like other diversities, women and females have been sorely underrepresented in clinical studies and non‐human animal research. Different strategies for inclusion have emerged.

**Objectives:**

To (i) identify what is known about sex and gender in resilience; (ii) consider key unknowns including the intersections of Sex and Gender with SSDH; (iii) provide recommendations for future inclusion of sex and gender as an intersecting factor in diverse populations’ resilience in aging and dementia.

**Method:**

Consolidating the talks of five experts in sex and gender and aging research and determining the themes emerging, we synthesize key recommendations for integrating sex and gender and SSDH.

**Results:**

Human diversity intersects with the multiple biologies of sex and the social worlds of gender. Including SSDH increases the complexity of analysis but leads to more precise understandings of aging. Lessons learned from women's health research teach us that there is a myriad of biologies within a single classification (eg., number of children, type of menopause) leading to multiple aging trajectories requiring that we not restrict ourselves to large cohort studies but instead, dive deeply into single groups.

**Conclusion:**

Sex and gender research provides helpful recommendations for both studying diverse populations as well as integrating sex and gender in diversity in resilience research.

